# ^Context or arousal? Function of drumming in Mongolian gerbils (*Meriones unguiculatus*)^

**DOI:** 10.1186/s12983-024-00542-2

**Published:** 2024-09-10

**Authors:** Yara Silberstein, Janina Büntge, Felix Felmy, Marina Scheumann

**Affiliations:** Institute of Zoology, University of Veterinary Medicine Hannover, Bünteweg 17, 30559 Hannover, Germany

**Keywords:** Drumming, Communication, Arousal, Alarm, Dominance, Mating, Gerbil, Vocalizations, Body posture

## Abstract

Drumming is a non-vocal auditory display producing airborne as well as seismic vibrations by tapping body extremities on a surface. It is mostly described as an alarm signal but is also discussed to signal dominance or mating quality. To clarify the function of drumming in Mongolian gerbils (*Meriones unguiculatus)*, we compared the occurrence of drumming during predator, opposite-sex and same-sex encounters. We tested 48 captive Mongolian gerbils (*Meriones unguiculatus*) in three experiments. In predator experiments, subjects were exposed alone or with their cagemate to aerial and terrestrial predator dummies. In social encounter experiments, familiar and unfamiliar male–female dyads and same-sex dyads were confronted. For the same-sex encounters, a dominance index was calculated for each subject based on the number of won and lost conflicts. Drumming and drumming-call combinations were counted, and a multi-parametric sound analysis was performed. In all experiments drumming and drumming-call combinations occurred. In predator experiments, more subjects drummed when confronted with the predator stimulus than in the habituation phase. In social encounter experiments, more subjects drummed when facing an unfamiliar than a familiar conspecific. In addition, the accompanying call type and body posture of the sender differed between experiments. Thus, we suggest that whereas drumming signals an increased arousal state of the sender, the accompanying call type and the body posture signal context specific information.

## Background

Drumming is a non-vocal form of acoustic communication where sounds are produced by stomping body parts or objects on a surface e.g., the ground or other body parts (e.g., [1–7]). Thereby, acoustic signals but also seismic vibrations are generated [8]. Drumming is found in several mammalian orders (e.g., Marsupialia: [9], Artiodactyla: [1], Carnivora: [10], Primates: [4, 6, 11], Rodentia: [12–14]) and can be accompanied by vocalizations as well as specific body postures and facial gestures, serving as a multimodal communication signal [7]. A neurobiological study in macaque monkeys showed that the auditory perception of drumming and vocalizations overlap in the caudal auditory cortex and the amygdala [15], suggesting a common origin of vocal and non-vocal behavior. Thus, the multisensory integration of drumming, vocalizations, and body postures can be expected to enhance the information encoded.

Randall [8] suggests several functions of drumming behavior. Drumming is mostly described as an anti-predator behavior, either to inform the predator that it has been detected (e.g., [13, 14]), or to warn group members (e.g., [7, 16]). Thus, drumming as a warning signal can be considered as a fitness advantage, since it prevents group members from predator attacks. Drumming in predator contexts can be accompanied by alarm calls [17]. In general, alarm calls can provide additional detailed information about the predator, like the predation type (aerial, terrestrial) or the urgency to respond inducing the respective anti-predator behavior (e.g., [18–22]). Drumming has also been observed in relation to the dominance status of the sender, either as a territorial signal to defend the own or claim territory (e.g., [23, 24]), or as a social dominance signal to reflect dominance hierarchy within the group [8]. Additionally, drumming has been documented before, during, and after mating [8]. Male kangaroo rats, for example, drum in front of other males to compete for access to estrous females, but also drum during the courtship ritual alongside the female [7]. Further, male African mole rats drum between mating sessions to signal readiness for mating [24]. Alternatively, it was suggested that drumming behavior is related to the affective state of the sender e.g., as a displacement behavior in situations of increased negative arousal [8, 25]. To sum up, four main functions of drumming have been suggested: (1) to warn conspecifics or to signal predator detection (anti-predator behavior), (2) to signal social dominance, (3) to coordinate mating interactions, or (4) to signal the arousal state of the sender. The Mongolian gerbil (*Meriones unguiculatus*) is a social species, for which drumming with the hind limbs has been described in alarm, mating, and dominance context (e.g., [26, 27]). Therefore, the Mongolian gerbil is a suited animal model to investigate the context-specificity of drumming and its associated vocal and visual displays.

Mongolian gerbils live in social groups of 2–17 individuals in underground burrows with several exits [28, 29]. A group consists of one dominant monogamous couple and their offspring, which are highly territorial and establish a dominance hierarchy within the group [30]. Adult males (80–130 g) can be slightly heavier than females (60–100 g) [31]. Females have polyestrous cycles, after they reach sexual maturity with 10–12 weeks [28]. Natural predators of Mongolian gerbils are snakes, foxes, martens (terrestrial predator) as well as owls and buzzards (aerial predators) [26, 29]. The vocal repertoire of Mongolian gerbils and the hearing range reach from low frequency to the ultrasonic range (100 Hz–60 kHz) [26, 32, 33]. In the ultrasonic range different call types have already been described [26, 33]. Kobayasi and Riquimaroux [33] described several vocalizations of Mongolian gerbils without allocating a context to them e.g., arched frequency modulated syllables (AFMs), short bent upward frequency modulated syllables (bUFMs), upward sinusoidal frequency modulated syllables (uSFMs), or downward frequency modulated syllables (DFM). During predator context, drumming is suggested to be accompanied by alarm calls and produced in an upright body posture, but quantitative data is lacking [26, 34]. Furthermore, drumming was observed during sexual behavior between males and females [27, 35] but also during aggressive interactions of same-sex conspecifics [27]. Thus, the function of drumming behavior in Mongolian gerbils is still unclear and quantitative data on the occurrence as well as on the acoustic structure of drumming and their associated vocalizations is missing.

In this study, we aim to investigate whether drumming of Mongolian gerbils functions as anti-predator behavior, dominance, mating, or arousal signal. To investigate anti-predator behavior, we exposed Mongolian gerbils alone or with their cagemate to predator dummies (predation experiment). To test whether drumming functions as a mating signal, we confronted familiar and unfamiliar male–female dyads (opposite-sex encounters). To investigate its function as a dominance signal, we confronted familiar and unfamiliar same-sex dyads (same-sex encounters). If drumming functions as anti-predator behavior, we expect that drumming occurs only in predation experiments but not during same-sex or opposite-sex encounters. If it functions to alert conspecifics, subjects are expected to drum more when tested with an audience than when tested alone. If drumming functions as a mating signal, we expect that drumming occurs exclusively during opposite-sex encounters. If drumming functions as a dominance signal, we expect that it occurs only during social encounter experiments but not during predation experiments. Thereby, the number of drummings should correlate with the dominance index of the sender. If drumming behavior would appear in all three experiments, we suggest that drumming behavior encodes general arousal of the sender. To test whether drumming functions as a multimodal signal, we further investigate whether potential associated call types or body posture change with its function.

## Methods

### Animals and housing

We conducted three experiments with 48 adult gerbils ranging between two to six months of age. The study was conducted in accordance with the European Community regulations about the protection of experimental animals and the guidelines of the German Animal Welfare Act, and approved by the Niedersächsisches Landesamt für Verbraucherschutz und Lebensmittelsicherheit, Germany (protocol code 33.8-42502-04-20/3372 and date of approval 2020-05-28). In the predator experiments, we tested 30 individuals (16♂, 14♀), 44 individuals in the opposite-sex (22♂, 22♀), and 26 individuals in the same-sex encounter experiments (12♂, 14♀). All animals were born at the breeding colony of the Institute of Zoology of the University of Veterinary Medicine Hannover, Foundation. The animals lived in same-sex groups of up to five individuals or as breeding pairs. They were housed in macrolan cages (610 × 435 × 215 mm), lined with approximately seven cm of wood shavings and equipped with a wooden hiding/nesting box. Pellets (SSNIFF Spezialdiäten GmbH, Soest, Germany, Complete diet for gerbils—1 mm) and water were offered ad libitum. Every other day a few pieces of vegetable and a tissue for nest building were offered. The breeding pairs and same-sex groups were housed in two different rooms with a humidity of 47 ± 7%, a temperature of 22 ± 2 °C, and a light/dark cycle of 12:12 h (lights on at 07:00).

### Experimental set up

All experiments were carried out in a semi-soundproof chamber to prevent interfering noises. The set up consisted of a wire mesh cage (100 × 40 × 30 cm) divided into two parts (50 × 40 × 30 cm each) connected by a small mechanical opaque door, which could be opened and closed from the outside of the chamber. The cage was positioned on a table equipped with a towel and a thin layer of litter to minimize walking sounds. During the experiments, the animals had access to food, water, and their home-nesting box (predator experiments) or the transport box (encounter experiments).

Sound was recorded using two microphones (MKH 8020; frequency range 10 Hz–70 kHz, Sennheiser electronic GmbH and Co. KG, Wedemark, Germany) per cage part, connected to a Zoom F4 or F6 Multitrack Field Recorder (K.K. Zoom corporation, Chiyoda, Tokyo, Japan) with a sampling rate set to 192 kHz. Additionally, video material was recorded using four digital cameras E1 (Reolink, Compton, CA, USA), which were localized laterally and above each cage part. The cameras were connected to a Synology surveillance system (Synology Inc., New Taipei City, Taiwan) and linked to monitors outside the chamber. Thus, the experimenter monitored all experiments from outside the chamber.

### Experimental Procedure

#### Predator experiments

Subjects were tested alone and with their cagemate in three different conditions: (A) simulating a terrestrial predator, (B) simulating an aerial predator, or (C) a control without predator presentation. For the terrestrial predator stimulus, a remote-controlled robot ZOOB BuilderZ with dimensions of 25 × 17 × 17 cm (excl. antenna) and for the aerial predator stimulus, a wooden bird (31 × 62 × 7 cm) moving on a string above the experimental cage were used.

For each experimental trial, a subject was placed alone or with its cagemate in one half of the cage. The experimental trial started with a 15 min habituation followed by a 15 min confrontation phase. Between the habituation and confrontation phase the experimenter entered the room to prepare the predator simulation. For the terrestrial predator, the experimenter removed a cardboard hiding the robot in the other half of the cage and moved it for 15–30 s every three to five minutes from outside the chamber. For the aerial predator, the wooden bird slid from the back of the chamber above the cage for ten seconds every three minutes by pulling a string attached to it from outside the chamber. Afterwards, the individuals were brought back to their home cages. Each subject was tested six times (single-terrestrial, single-aerial, single-control, cagemate-terrestrial, cagemate-aerial, cagemate-control). The order of the conditions was pseudo-randomized.

#### Encounter experiments

In the social encounter experiments, opposite- or same-sex dyads were confronted with familiar (= subjects were housed together in the same cage) or unfamiliar conspecifics (= subjects were not housed in the same cage).

At the beginning of the experimental trial, each subject was placed in opposite sides of the cage and observed for 15 min with the door closed. Afterwards, the door was opened, and the dyad was observed for further 15 min.

For the opposite-sex encounters, we used different subjects for familiar and unfamiliar encounters, because familiar encounters consisted of breeding pairs, which should not be stressed by unfamiliar social encounters. Thus, for familiar encounters nine breeding pairs and for unfamiliar encounters 13 dyads of same-sex housed subjects were used. For the same-sex encounters, same-sex housed subjects participated in unfamiliar and familiar encounters. The order of the familiar, unfamiliar, same-, or opposite-sex encounters was pseudo-randomized. Additionally, the order of predator and social encounter experiments was pseudo-randomized.

### Audio and video analysis

To determine the number of drumming, all audio files were screened auditory and visually using spectrograms generated by the software Audacity (Free Software Foundation, Inc., Version 2.1.2, Boston, MA, USA, www.audacityteam.org). Additionally, drummings were confirmed by checking the video data using VLC Media player (VideoLAN Organization, Version 3.0.1. Vetinari, Paris, France) and the sender as well as its body posture (Upright—subject stands on his hind legs and the fore legs are in the air; Non-upright—all four legs of the subjects are in contact with the ground or the grid or the subject hides in the box) was noted. A drumming was defined as a sequence of rapid broadband pulses produced by rapid movements of the hind legs of the animal. Additionally, it was noted whether the drumming was accompanied by a vocalization. A vocalization was counted if the vocalization overlapped the first up to the second pulse of the drumming.

To determine the dominance relationship between subjects in the encounter experiments, video data was analyzed using Observer XT (Noldus Information Technology, Version 12.5, Wageningen, Netherlands). First, socio-negative interactions were identified, and the winner or loser of each interaction was noted. Winners were defined as the subject which chased the dyad partner, while losers were defined as the subject which was chased by the dyad partner. Second, a dominance index between 1 (dominant) and -1 (subdominant) was calculated for every individual in each experiment ($\frac{No.\ of\ won\ conflicts - No.\ of\ lost\ conflicts}{No.\ of\ won\ conflicts + No.\ of\ lost\ conflicts}$).

For the description of the acoustic parameters of drummings, we randomly selected 10 drummings, if applicable, for each individual per phase per experimental condition per experiment. For the drummings, the total duration, number of pulses, and the duration from the first to second pulse were determined manually using spectrograms generated in BatSound (FFT 2048, time window: 2000 ms). Additionally, the pulse rate was calculated as no. of pulses divided by total duration (Table 1). In total we measured 299 drummings, consisting of 87 in the predator experiments (n = 9 individuals), 160 in the opposite-sex encounters (n = 17 individuals), and 52 in the same-sex encounters (n = 5 individuals).

**Table 1:** Description of measured acoustic parameters of drumming and vocalizations accompanying drumming

Acoustic parameter	Abb.	Definition
**Drumming**		
Drumming duration [ms]	D_Dur	Time between the first and last pulse of a drumming
Number of pulses	no. pulses	Amount of visible/hearable pulses in a drumming
Duration from first to second pulse [ms]	first_second	Time between the onset of the first to the onset of the second pulse
Pulse rate	P_rate	No. of pulses divided by the total duration of the drumming
**Vocalizations**		
*Time-related parameters*		
Call duration [ms]	C_Dur	Time between the onset and the offset of a vocalization
Time of minimum fundamental frequency [ms]	TimeminF0	Time between the onset and the time point of minimum fundamental frequency of a vocalization
Time of maximum fundamental frequency [ms]	TimemaxF0	Time between the onset and the time point of maximum fundamental frequency of a vocalization
*Source-related parameters*		
Minimum fundamental frequency [kHz]	MinF0	Lowest value of the fundamental frequency across all time frames of a vocalization
Maximum fundamental frequency [kHz]	MaxF0	Highest value of the fundamental frequency across all time frames of a vocalization
Bandwidth [kHz]	BandF0	MaxF0—MinF0
Mean fundamental frequency [kHz]	MeanF0	Mean fundamental frequency of a vocalization calculated across all time frames of a vocalization
Standard deviation of fundamental frequency [kHz]	SDF0	Standard deviation of the fundamental frequency of a vocalization calculated across all time frames of a vocalization
Meanslope [kHz/s]	SlopeF0	Mean absolute slope of the fundamental frequency calculated as the sum of the absolute difference of the F0 of two consecutive time frames
*Filter-related parameters*		
Center of gravity [kHz]	CoG	Mean frequency of the spectrum of a vocalization weighted by the amplitude of a vocalization
Standard deviation of CoG [kHz]	SD	Standard deviation of the CoG measuring the deviation of frequency values from the CoG of a vocalization
Skewness	Ske	Difference between the spectral distribution below and above the CoG of a vocalization
Kurtosis	Kur	Difference between the spectral distribution around the CoG from a Gaussian distribution of a vocalization
*Tonality-related parameters*		
Voiced percentage [%]	Voiced	Percentage of voiced time frames of a vocalization
Harmonics-to-noise-ratio [dB]	Hnr	Ratio between the periodic (harmonic part) and aperiodic (noise) components of a vocalization
Wiener entropy [dB]	Entropy	Ratio of geometric to arithmetic energy of a vocalization

Time frame for the analysis of source-related vocalization parameters = 3 ms; *Abb.* Abbreviations

**Fig. 1:**
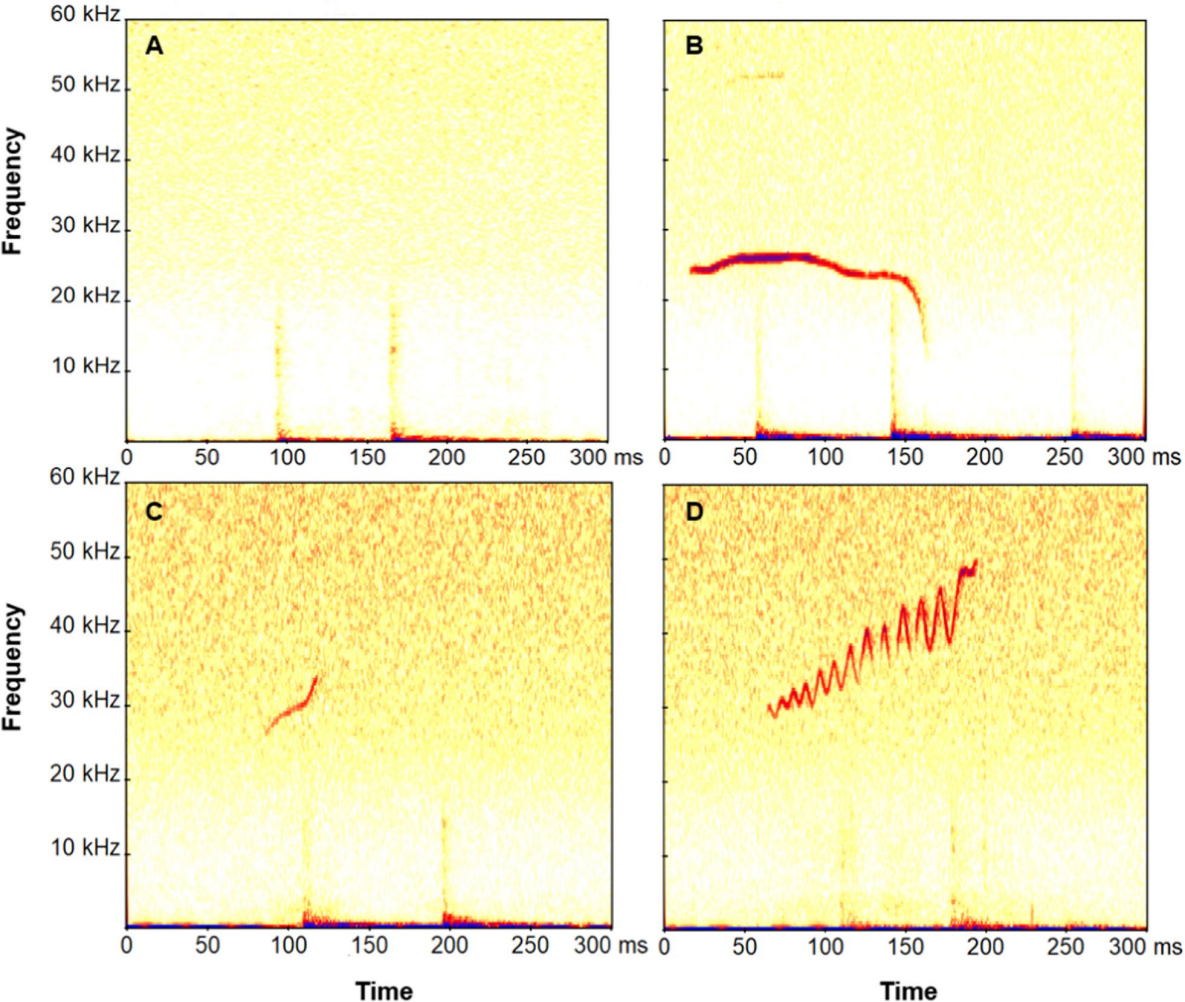
Spectograms of **A** drumming without vocalization, **B** drumming with AFMs, **C** drumming with bUFMs, and **D** drumming with uSFMs; *AFMs* arched frequency modulated syllables, *bUFMs* short bent upward frequency modulated syllable, *uSFMs* upward sinusoidal frequency modulated syllables

For drumming associated vocalizations, we performed a multi-parametric sound analysis with the program PRAAT (Version 6.1.12, Phonetic Sciences, University of Amsterdam, The Netherlands; [36]) combined with GSU Praat Tools 1.9 scripts [37] on all vocalizations of good quality (high signal-to-noise-ratio, not clipped). We pre-processed the audio files by band-pass-filtering them (500–100,000 Hz) to improve signal-to-noise ratio. We measured 16 different acoustic parameters: call duration (C_Dur), time of minimum fundamental frequency (TimeminF0), time of maximum fundamental frequency (TimemaxF0), minimum fundamental frequency (MinF0), maximum fundamental frequency (MaxF0), bandwidth (BandF0), mean fundamental frequency (MeanF0), standard deviation of fundamental frequency (SDF0), meanslope (SlopeF0), center of gravity (CoG), standard deviation of the center of gravity (SD), skewness (Ske), kurtosis (Kur), percentage of voiced frames (Voiced), harmonics-to-noise ratio (Hnr), and wiener entropy (Entropy; Table 1). We used the To Pitch (cc) command to track the contour of the fundamental frequency and compared it to the spectrogram to correct the tracking manually if necessary. Additionally, we performed a visual classification of the vocalizations using call types (AFMs; bUFMs; uSFMs) described by Kobayasi & Riquimaroux [33] (Fig. 1). As the number of drumming varied strongly between individuals, we balanced our dataset by randomly selecting 10 drummings with good quality of each individual per phase per experimental condition per experiment for the statistical analysis of the acoustic parameters. In total, we measured 320 vocalizations, consisting of 189 vocalizations in the predator experiments (n = 8 individuals), 52 vocalizations in the opposite-sex experiments (n = 11 individuals), 79 vocalizations in the same-sex experiments (n = 3 individuals).

### Statistical analysis

To investigate if there was an effect on the drumming occurrence (yes/no), we performed binomial Bayesian Generalized linear Mixed-Effects Models (bGLMM) using the “blme” package in R. The bGLMM models are recommended for datasets containing complete separation, which was the case for some predictors in our data set [38]. The bglmer function requires to specify a fixed effect and random effect priors. For predictors with complete separation a weak, zero mean normal prior equal to three standard deviations was applied [39]. The random effect prior was set to NULL applying flat priors over all parameters [38]. We used drumming occurrence as test factor and controlled for individual as random factor. For the predator experiment, we tested the predictors Audience (single/with cagemate), Phase (habituation/confrontation), Sex (male/female) and Condition (predator/control). We did not differentiate between terrestrial and aerial predator, because a preliminary analysis revealed no difference in drumming occurrence between predator types (Est. = − 0.02, SE = 0.73, Z = − 0.02, p = 0.983). For opposite-sex and same-sex encounter experiments, we used Familiarity (unfamiliar/familiar), Sex (female/male) and Door (open/closed) as predictors. Additionally, the dominance index was used in the model for the same-sex encounter experiments. We used the model with the main terms as a basic model (e.g., predator experiment: Occurrence ~ Audience + Sex + Phase + Condition + (1|Individual)). To disclose an interaction between our predictor variables, we added each possible two-way interaction separately to the basic model. After that, we compared the models containing a two-way interaction with the basic model using Wald test statistics (“anova” command). If the basic model and the interaction model did not differ significantly, we used the basic model as final model. In the case the models differed from each other, we used the model with the respective interaction as final model. In the result section, we will only report on final models.

To investigate if there was an effect on the number of drummings (count data) and the number of drumming-call combinations produced, we performed General Linear Mixed Models (GLMM) using a Template Model Builder (TMB) [40]. We used the generalized poisson (genpois) distribution [41] for all models and checked the model assumptions using the DHARMa package (KS-, dispersion- and outlier test, residuals versus predicted and zero-inflation test; [42]) as recommended by Santon et al. [40]. The number of drummings or number of drumming-call combinations per experimental trial were used as test variables and we added the same predictors and random factors as described for the basic model of drumming occurrence. For four drumming-call combinations, we could not determine the caller, because both interaction partners drummed at the same time. Therefore, we excluded them from further analyses involving the accompanying vocalizations.

Comparing drumming behavior across experiments, we compared the confrontation phase of the predator experiments (except for the control condition) with the unfamiliar condition of the opposite- and same-sex social encounter experiments. In this analysis, 30 subjects were included from which 19 subjects contributed with data for all experiments. Due to technical problems, we had to exclude four subjects for the same-sex and two for the opposite-sex experiments. Further two dyads had to be excluded from the opposite-sex experiments, because the experimenter had to intervene to stop aggressive interactions for welfare reasons. Two subjects participated only in the predator condition because of health reasons. To compare the experiments, we calculated a binomial bGLMM model for drumming occurrence and a glmmTMB for number of drummings and number of drumming-call combinations using Experiment as predictor (predator/opposite-sex/same-sex) and individual as random factor. Since experiments contained three levels, we calculated an ANOVA for the final model using the “car” package. For pairwise comparison we used the lsmeans function of the “emmeans” package.

To investigate if there was an effect on the acoustic parameters of drummings, we performed Linear Mixed Models (LMM) for each parameter and experiment separately. The models consisted of the acoustic parameter as test factor as well as the same predictor and random factors as described for the basic model of drumming occurrence (e.g., LME predator experiments: Acoustic parameter ~ Audience + Sex + Phase + Condition, random = Individual).

To investigate whether our visual classification of call types can be mathematically confirmed, we performed an independent discriminant function analysis. We standardized the acoustic parameters using z-transformation and calculated pairwise Pearson correlations to test the acoustic parameters for independence. If the correlation coefficient was higher or equal than 0.7, only one of the two parameters was included in the following analysis. To account for repeated measurement of the same individuals, the discriminant analysis was confirmed by a permutated discriminant function analysis [43].

We classified the drumming posture in two categories: “upright” and “non-upright” posture. To investigate whether drumming posture was affected by the type of experiment or whether a vocalization was overlapping the drumming, we performed a binomial bGLMM. We calculated a basic model containing body posture (Upright posture: yes/no) as test variable, experiment (predator/opposite-sex/same-sex) and call occurrence (yes/no) as predictor variable while controlling for individual. Additionally, we calculated a full model including the interaction term between experiment*call occurrence. If the interaction was not significant and the full model did not differ from the model without the interaction term tested by using Wald statistics, we report on the basic model. For the drumming accompanied by vocalizations, we further calculated a binomial bGLMM model using call type as predictor variable while controlling for individual. We used the same fixed effects and random priors as described above.

For the statistical analysis R (Version 4.3.0) was used, accessed by RStudio (Version 2023.06.1–524.0); packages: bGLM—“blme” (1.0–5), “lme4 (1.1–34) as well as the optimizer “bobyqa”; GLMM-TMB—“Dharma” (0.4.6), “glmmTMB” (1.1.7); LME—“nlme” (3.1–162); Anova—“car” (3.1–2); lsmeans—“emmeans” (1.10.1); DFA—“DFA.CANCOR” (0.2.8), “MASS” (7.3–60.2); graphical illustrationns “ggplot2” (3.4.3).

## Results

### Drumming behavior

In all experimental conditions drummings were produced, resulting in a total of 1536 drummings produced by 19 animals. During the predator experiments, 354 drummings appeared (N = 9), 752 during the opposite-sex encounters (N = 17), and 430 during the same-sex experiments (N = 5). Only three individuals produced drummings in all three experiments.

**Fig. 2:**
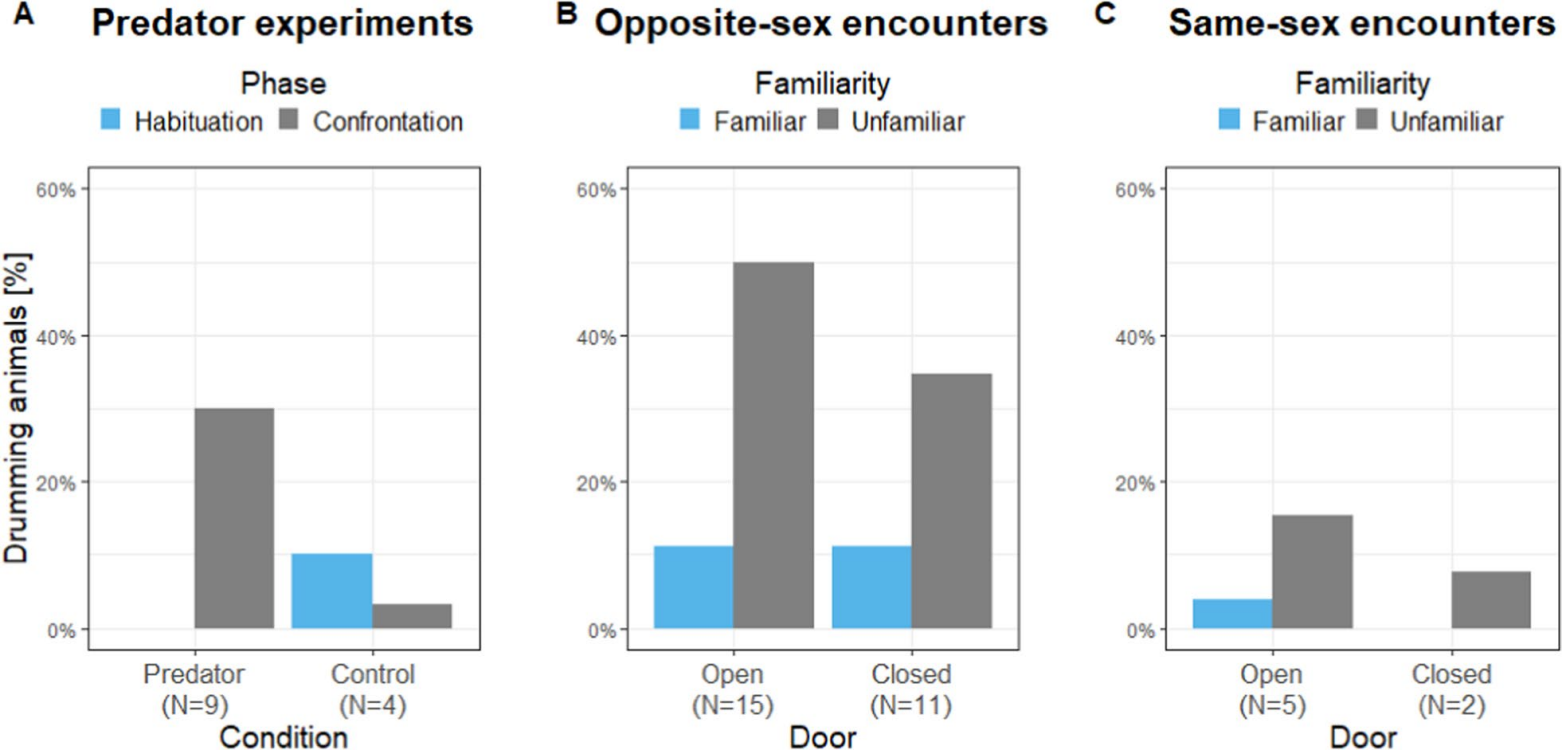
Individuals producing drumming comparing **A** the experimental phases of the predator experiments and the individuals’ familiarity in the **B** opposite-sex and **C** same-sex encounters; *N* number of drumming individuals

For the predator experiment, the final model for drumming occurrence contained a significant interaction of Condition and Phase (Est. = 3.85, SE = 1.62, Z = 2.38, *p* = 0.018; Table S1), whereas Audience and Sex of the animals had no significant effect on the drumming occurrence (Est. ≤ 0.58, SE ≤ 0.83, Z ≤ 0.70, *p* ≥ 0.485). Thus, in the control condition more animals produced drummings during the habituation versus the confrontation phase, whereas in the predator condition subjects produced drummings exclusively in the confrontation phase (Fig. 2A). For the number of drummings no effect of Condition, Audience, and Sex was found (Est. ≤ 0.58, SE ≤ 0.80, Z ≤ 0.73, *p* ≥ 0.468), but subjects drummed significantly more often in the confrontation than habituation phase (Est. = 1.30, SE = 0.66, Z = 1.97, *p* = 0.049).

For the opposite-sex and same-sex encounters, the final models consisted only of main terms showing an effect of Familiarity on drumming occurrence for opposite-sex encounters (Est. = 3.99, SE = 1.81, Z = 2.20, *p* = 0.028) and a trend for same-sex drumming occurrence (Est. = 6.09, SE = 3.21, Z = 1.90, *p* = 0.058; Table S1), whereas the other predictors were not significant (Est. ≤ 2.97, SE ≤ 3.42, Z ≤ 1.49, *p* ≥ 0.135). Additionally, the number of drummigs differed between familiar and unfamiliar conditions for both, opposite- and same-sex encounters (Est. ≤ 2.88, SE ≤ 1.41, Z ≥ 2.04, *p* ≤ 0.041), whereas the other predictors were not significant (Est. ≤ 1.34, SE ≤ 0.96, Z ≤ 1.45, *p* ≥ 0.148). Thus, drumming occurrence (Fig. 2B, C) and drumming rate were higher in unfamiliar compared to familiar dyads.

For the acoustic parameters, drumming duration was affected by Condition and Audience in the predator experiment and by Familiarity in the same-sex experiment. In the predator experiments, drumming duration was significantly longer when subjects were tested alone compared to be tested with a conspecific (Est. = 95.94, SE = 41.58, t = 2.31, *p* = 0.024; Table S2) and longer when exposed to the predator than control condition (Est. = 313.32, SE = 69.78, t = 4.49, *p* < 0.001). In same-sex encounters, but not in opposite-sex encounters, drumming duration was longer when exposed to unfamiliar compared to familiar conspecifics (Est. = 135.11, SE = 53.50, t = 2.53, *p* = 0.015). Furthermore, in predator experiments the pulse rate was higher when subjects were tested with a conspecific compared to be tested alone (Est. = − 0.25, SE = 0.09, t = − 2.64, *p* = 0.010).

Comparing the three experiments, the drumming occurrence and no. of drummings differed between experiments (χ^2^ ≥ 23.24, df = 2, *p* < 0.001). Thus, drumming occurrence and no. of drummings were higher in opposite-sex encounters compared to predator experiments or same-sex encounters (occurrence: Est. ≥ 1.83, SE≤ 0.66, t ≥ 2.77, *p* ≤ 0.016; no. of drummings: Est. ≥ 1.44, SE ≤ 0.47, t ≥ 3.04, *p* ≤ 0.007). Acoustic parameters did not differ significantly between experiments (χ^2^ ≤ 1.85, df = 2, *p* ≥ 0.398).

### Accompanying vocalizations

Vocalizations accompanying drumming were produced in all experimental conditions. Thereby, 27% (n = 420; 16 subjects) of the drummings were overlapped by vocalizations. Thus, in the predator experiments 54% (n = 190, N = 8), in the opposite-sex encounters 16% (n = 124; N = 13), and in the same-sex experiments 25% (n = 106, N = 4) of drummings were overlapped by vocalizations. For only one individual drumming-call combinations were recorded in all three experiments. In the predator experiments the number of drumming-call combinations tended to be higher in the confrontation compared to the habituation phase (Est. = 1.51, SE = 0.79, Z = 1.90, *p* = 0.058; Table S3), whereas there was no difference for Audience, Sex and Condition (Est. ≤ 0.67, SE ≤ 0.85, Z ≤ 0.85, *p* ≥ 0.397). For the opposite-sex encounters, the number of drumming-call combinations was significantly higher when exposed to unfamiliar versus familiar conspecifics (Est. = 2.15, SE = 0.96, Z = 2.23, *p* = 0.026) and showed a trend to be higher in males than females (Est. = − 1.44, SE = 0.83, Z = − 1.73, *p* = 0.084), whereas Door opening had no effect (Est. = 0.27, Se = 0.49, Z = 0.54, *p* = 0.586; Table S3). For the same-sex encounters, none of the predictors had an effect on the number of drumming-call combinations (Est. ≤ 2.43, SE ≤ 1.73, Z ≤ 1.58, *p* ≥ 0.115; Table S3). Finally, comparing the three experiments, the no. of drumming-call combinations differed between experiments (χ^2^ = 19.01, df = 2, *p* < 0.001), meaning more drumming-call combinations were uttered in the opposite-sex than in the predation context (Est. = 1.86, SE = 0.44, Z = 4.27, *p* < 0.001), but there was no difference between the social encounter experiments or same-sex encounters and the predator experiments (Est. ≤|1.05|, SE ≤ 0.57, Z ≤ 2.04, *p* ≥ 0.103).

**Fig. 3:**
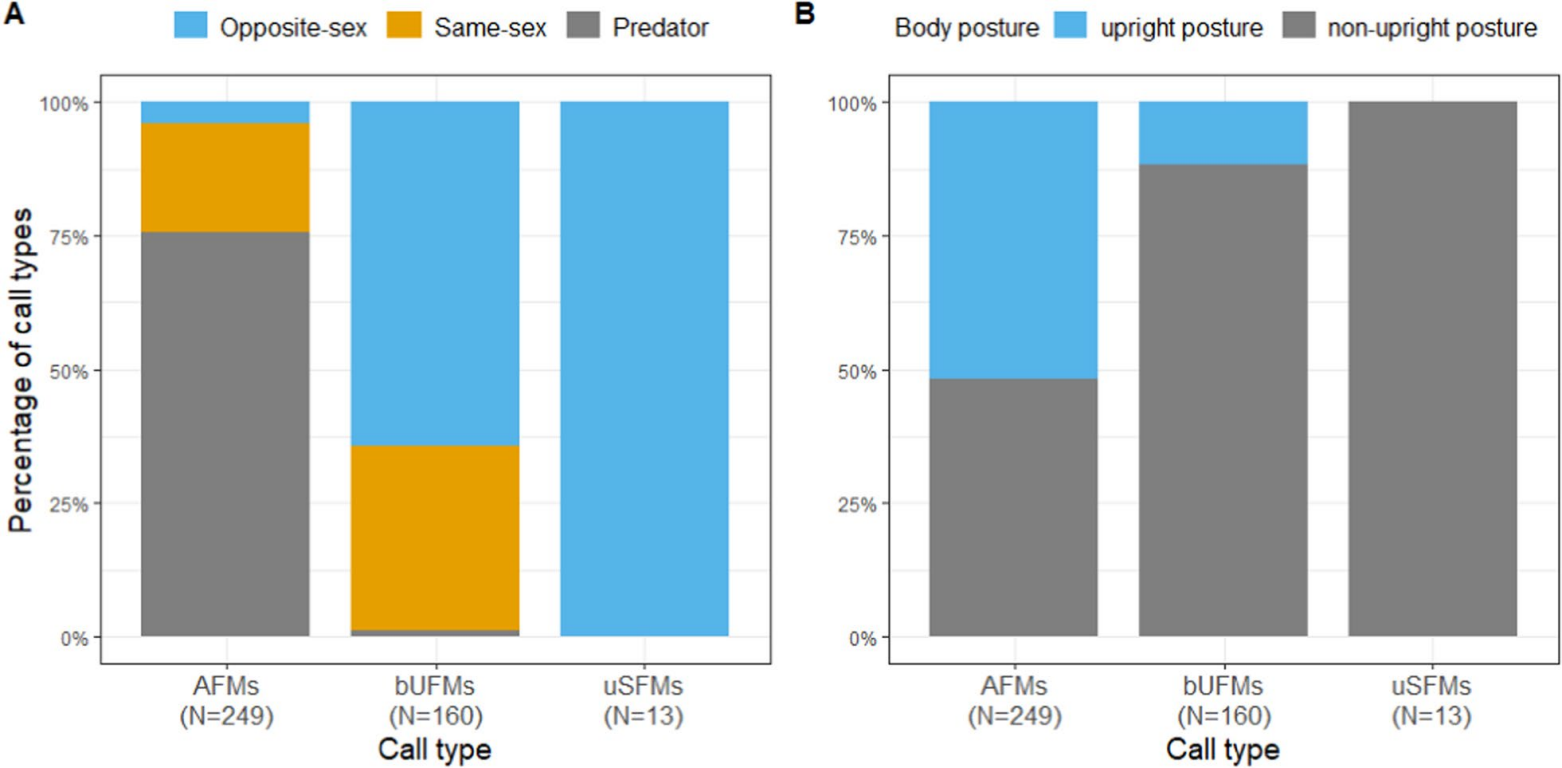
Barplot of the percentage of call types uttered **A** in the different experiments and **B** combined with an upright or non-upright body posture while drumming; *AFMs* arched frequency modulated syllables, *bUFMs* short bent upward frequency modulated syllable, *uSFMs* upward sinusoidal frequency modulated syllables; *N* number of drumming individuals

To investigate whether the call type of the accompanying vocalizations is context-specific, we performed a discriminant function analysis to confirm our visual classification using six non-correlating acoustic parameters of vocalizations: C_Dur, MinF0, MaxF0, SDF0, Ske and Entropy (see Table S4 for mean and standard deviation of the acoustic parameters). The DFA revealed that 98.1% of vocalizations (cross-validation: 97.8%) were correctly classified, which was confirmed by a pDFA controlling for individual (*p* ≤ 0.004). Additionally, the DFA revealed that 98.0% of the vocalizations (cross-validation: 97.5%) were correctly assigned to AFMs, 98.6% to bUFMs (cross-validation: 98.6%) and 100.0% to uSFMs (cross-validation: 100%). In the predation experiment, 188 of 190 vocalizations belong to the AFMs (Fig. 3A). In the opposite sex condition, 103 of 124 vocalizations belong to bUFMs. In the same-sex experiment, we recorded 51 AFMs, which were produced at one event where the experimenter had to enter the room to stop a conflict. Excluding these vocalizations, 100% of the vocalizations (N = 55) were bUFMs. Thus, in the predation contexts or in response to the experimenter AFMs accompanied drumming, whereas in social encounter experiments mainly bUFMs were recorded. Moreover, uSFMs occurred rarely but exclusively in the opposite-sex encounters.

### Body posture

Focusing on all drumming events, the body posture showed an interaction between the type of experiment and if a vocalization was associated or not (χ^2^ = 6.78, df = 2, *p* = 0.034). A breakdown analysis showed that in drumming-call combinations as well as in drummings without a vocalization the body posture differed depending on the experiment (χ^2^ ≥ 13.20, df = 2, *p* ≤ 0.001). Thus, during predator experiments an upright position was taken more often in drumming-call combinations than in the social encounters (Est. ≥ 3.27, SE ≤ 0.79, Z ≥ 4.44, *p* < 0.001). Moreover, the effect of call type on the occurrence of drumming-call combinations revealed that an upright posture occurred significantly more often when an AFMs overlapped the drumming than a bUFMs or uSFMs (χ^2^ = 29.41, df = 2, *p* < 0.001; Fig. 3B).

## Discussion

Our results show that Mongolian gerbils produce drumming in predator as well as in opposite-sex and same-sex encounter experiments. Thus, the results suggest that drumming is not context specific, but signals the arousal level of the sender. This is further supported by the finding that subjects also drummed when they were alone and more often during unfamiliar than familiar social encounters. In all three experiments, drumming-call combinations were observed. In addition, the structure of the vocalizations differed. Thus, in predation experiments mainly AFMs were produced, whereas in social encounters mainly bUFMs were recorded, and uSFMs occurred exclusively in the opposite-sex experiments. In addition, the body posture was adapted to the call type and experiment, showing an upright position during AFMs as well as in predator experiments, in comparison to other body postures. Therefore, we suggest that drumming-call combinations serve as a multimodal communication signal combining drumming, vocalizations, and the body posture of the sender.

Supporting the arousal hypothesis, drumming was observed in all three experiments. As drumming was not exclusively produced during predator contexts, the anti-predator hypothesis can be rejected. We found no effect of audience suggesting that drumming was not used to warn conspecifics [16]. The result that drumming occurred more during the confrontation than habituation phase in the predator condition confirms the assumptions of Ter-Mikaelian et al. [26] that drumming is used to signal predator detection. However, it can also be explained by a higher arousal of the sender when confronted with the predator. Since drumming also did not occur exclusively in the opposite-sex or same-sex encounters and drumming was not correlated with dominance index, the mating and social dominance hypotheses can be rejected, too. The fact that drumming occurred more often towards unfamiliar than familiar conspecifics also favors the arousal hypothesis, as an unfamiliar conspecific (potential intruder) induces a higher arousal level in the sender. The impact of arousal on drumming occurrence is in line with the suggestion of Randall [8] that drumming can also be a displacement behavior in situations of high arousal. Routtenberg & Kramis [25] suggested that drumming is related to negative arousal states. However, we found more drummings during opposite-sex encounters, suggesting that drumming is not only related to arousal states of negative valence but can also occur in arousal states of varying valence such as sexual arousal. The acoustic structure of vocalizations is suggested to encode arousal levels. According to Briefer [44], vocalizations of longer duration and fast repetition signal higher arousal states. Since it was found that drumming and vocalizations are processed in the same neurobiological structure in macaques [15], it can be assumed that arousal related changes in temporal parameters can also be applied to non-vocal acoustic communication sounds. In the predator experiments, we found that drummings were longer and had higher pulse rates when tested alone than tested with the cagemate. Thus, social isolation might result in a higher arousal state especially in highly social animals such as Mongolian gerbils. In the same-sex experiments, drumming duration was longer in unfamiliar compared to familiar social encounters. This further supports the expression of arousal, as it can be assumed that a potential unfamiliar intruder induces a higher arousal in the sender.

Whereas drumming seems to encode the arousal level of the subject, we suggest that the accompanying vocalizations and the associated body posture are context-specific. We identified three main call types which were confirmed by a discriminant function analysis, namely arched frequency modulated syllables (AFMs), short bent upward frequency modulated syllables (bUFMs), and upward sinusoidal frequency modulated syllables (uSFMs). AFMs occurred mainly during our predator experiments and match the alarm calls described by Ter-Mikaelian et al. [26] and Volodin et al. [34]. In contrast, bUFMs were produced mainly during social encounter experiments, which is in line with the function as contact call suggested by Ter-Mikaelian et al. [26]. Finally, uSFMs were only produced in opposite-sex encounters. Holman [45] recorded three different ultrasonic call types. Upsweep and modulated vocalizations were produced during the pre-copulatory and mounting phase and unmodulated vocalizations after ejaculation. The uSFMs vocalizations match the modulated vocalizations of Holman [45], suggested to function in the attraction of potential mating partners. The increased occurrence of drummings in opposite-sex encounters compared to predator experiments or same-sex encounters can also point to an increased sexual arousal when interacting with unfamiliar mating partners. Further studies linking drumming behavior to physiological measurements of arousal would be useful to validate the influence of arousal on drumming behavior.

Since we recorded drumming-alarm call combinations in the predator experiments even when the subject was tested alone, we can prove that alarm calls are associated with drummings. In the social encounter experiments, we have to admit that in contrast to the drumming, we were not able to allocate the ultrasonic vocalizations to the respective individual reliably, especially since vocalizations were often uttered when the animals were in close proximity to each other. Therefore, the overlaying bUFMs and uSFMs vocalizations might also be produced by the non-drumming dyad partner. Nevertheless, our results show clearly that AFMs combined with drumming are associated with predator contexts, whereas in social interactions no AFMs were combined with drumming. Interestingly, not all drummings were overlaid with vocalizations. Thus, for the predator experiments, for example, only 54% of the drummings showed a vocalization. This suggests a stimulus depended individual threshold, which would explain why only three individuals drummed in all three experiments, and the lower overall drumming occurrence throughout individuals (max. 50% of the individuals drummed; Fig. 2). The low responsiveness might also be an effect of laboratory housing. Captive gerbils are very curious and even if our gerbils are not regularly handled, they approach new objects/arenas very quickly, sniffing and touching the hand of the experimenter or entering the transport box voluntarily without extensive training. Thus, individual experience or personality may influence the general lower arousal levels. Thereby, it could be assumed that the threshold inducing drumming of the hind limbs is lower than the threshold to elicit vocalizations. Nevertheless, since it seems that drummings can be associated with different call types depending on the context, it can also be assumed that two different neuronal patterns for the motoric control of the hind limbs and the larynx are coupled. Further studies are needed to clarify the neuronal control of the vocal and non-vocal pathways of drumming-call combinations.

Concerning the body posture while drumming, we found an effect of the experiment and the associated call type. During predator experiments, individuals drummed more often in an upright posture and AFMs were the most common associated call type. In contrast, during social encounter experiments drummings were mostly produced in a sitting or running position associated with bUFMs and uSFMs. This matches the observations of Ter-Mikaelian et al. [26], who reported that alarm calls were uttered using an alarm posture, whereas drumming in sitting postures was used during social interactions. However, the alarm posture might not be an information for conspecifics, as Mongolian gerbils live in burrows [46] and thereby, do not see the alarm posture when they are in the burrow. Instead, the sound of the drumming might be more informative, as the sound is canalized in the burrow through a great distance [47]. In this regard, it is more likely that gerbils try to get a better view on the environment when standing in an upward position, e.g., looking for predators [17, 48]. However, during social interactions drumming occurred when facing each other. Thus, when encountering conspecifics, body posture seems to be a further signal for context-specificity. To clarify the function of posture, video playbacks might help to estimate the importance of drumming posture.

## Conclusions

In conclusion, our results suggest that while drumming signals the arousal state of the sender, overlaying vocalizations as well as the respective body posture can encode context-specificity. Thus, drumming in combination with vocalizations and body posture serves as a multimodal signal to increase the encoded information. To understand the interplay and the impact of the different sensory information**,** further experimental studies are needed e.g., video playbacks. Additionally, the investigation of the neuronal control of the different motoric units (hind limb, larynx) would shed light on the coupling of multiple sensory channels resulting in multimodal signals.

## Supplementary Information

Additional file 1

## Data Availability

Raw data is available on the data repository Zotero 10.5281/zenodo.13325012. Audio and video data are stored at the Institute of Zoology and are available on reasonable request.
