# Supplementary material for: Context or arousal? Function of drumming in Mongolian gerbils (Meriones unguiculatus)
Source: Front Zool. 2024 Sep 10;21:22. doi: 10.1186/s12983-024-00542-2 (PMC11386350; doi:10.1186/s12983-024-00542-2)
Supplement: Supplementary file 1 — Additional file 1 [file 12983_2024_542_MOESM1_ESM.docx]

Supplementary materials for

**Context or arousal? Function of drumming in Mongolian gerbils (*Meriones unguiculatus*)**

Yara Silberstein et al.

*Corresponding author. Email: [yara.silberstein@gmail.com](mailto:yara.silberstein@gmail.com)

This PDF file contains:

Tables S1 to S4**Table S1.**
Results of the bGLMM models testing the effects for drumming occurrence in predator, opposite-sex encounter and same-sex encounter experiments, as well as the results of GLMM models testing the effects for the amount of drumming produced in predator, opposite-sex encounter and same-sex encounter experiments; SE: standard error; Bold: p < 0.05

| **Drumming occurrence** | | | | |
| --- | --- | --- | --- | --- |
| Predictor | Estimate | SE | z-value | p-value |
| **Predator experiments (df = 30)** | | | | |
| Condition_predator | -2.72 | 1.49 | -1.83 | 0.067 |
| Sex_female | 0.58 | 0.83 | 0.70 | 0.485 |
| Phase_confrontation | -0.64 | 1.04 | -0.62 | 0.534 |
| Audience_single | 0.18 | 0.60 | 0.30 | 0.764 |
| Condition_predator*Phase_confrontation | 3.85 | 1.62 | 2.38 | **0.018** |
| **Opposite-sex encounters (df = 44)** | | | | |
| Familiarity_unfamiliar | 3.99 | 1.81 | 2.20 | **0.028** |
| Sex_female | -0.98 | 1.44 | -0.68 | 0.494 |
| Door_closed | -1.13 | 0.84 | -1.35 | 0.176 |
| **Same-sex encounters (df = 20)** | | | | |
| Familiarity_unfamiliar | 6.09 | 3.21 | 1.90 | 0.058 |
| Dyad_female-female | 0.32 | 3.42 | 0.09 | 0.926 |
| Door_closed | -2.97 | 1.99 | -1.49 | 0.135 |
| Dominance index | 2.23 | 1.72 | 1.30 | 0.194 |
| **No. of drumming** | | | | |
| Predictor | Estimate | SE | z-value | p-value |
| **Predator experiments (df = 354)** | | | | |
| Condition_predator | 0.08 | 0.61 | 0.13 | 0.898 |
| Sex_female | 0.58 | 0.80 | 0.73 | 0.468 |
| Phase_confrontation | 1.30 | 0.66 | 1.97 | **0.049** |
| Audience_single | 0.08 | 0.56 | 0.14 | 0.889 |
| **Opposite-sex encounters (df = 752)** | | | | |
| Familiarity_unfamiliar | 2.55 | 0.94 | 2.70 | **0.007** |
| Sex_female | -0.94 | 0.82 | -1.15 | 0.251 |
| Door_closed | 0.17 | 0.43 | 0.39 | 0.697 |
| **Same-sex encounters (df = 430)** | | | | |
| Familiarity_unfamiliar | 2.88 | 1.41 | 2.04 | **0.041** |
| Dyad_female-female | 0.09 | 1.64 | 0.05 | 0.958 |
| Door_closed | -1.04 | 0.96 | -1.09 | 0.274 |
| Dominance index | 1.34 | 0.93 | 1.45 | 0.148 |

**Table S2.**
Results of the LMM models testing the effects for drumming acoustic parameters in predator, opposite-sex encounter and same-sex encounter experiments; SE: standard error; Bold: p < 0.05

| **Drumming acoustic parameters** | | | | | |
| --- | --- | --- | --- | --- | --- |
| **Predator experiments** | | | | | |
| Predictor | Estimate | SE | df | t-value | p-value |
| **Duration** | | | | | |
| Condition_predator | 313.32 | 69.78 | 76 | 4.49 | **0.000** |
| Sex_male | 19.51 | 110.14 | 6 | 0.18 | 0.865 |
| Phase_confrontation | -32.21 | 43.92 | 76 | -0.73 | 0.466 |
| Audience_single | 95.94 | 41.58 | 76 | 2.31 | **0.024** |
| **Pulse rate** | | | | | |
| Condition_predator | 0.16 | 0.19 | 76 | 0.83 | 0.412 |
| Sex_male | 0.17 | 0.10 | 6 | 1.76 | 0.129 |
| Phase_confrontation | 0.06 | 0.22 | 76 | 0.29 | 0.775 |
| Audience_single | -0.25 | 0.09 | 76 | -2.64 | **0.010** |
| **First to second pulse** | | | | | |
| Condition_predator | -3.36 | 4.83 | 76 | -0.69 | 0.489 |
| Sex_male | -1.01 | 4.32 | 6 | -0.24 | 0.822 |
| Phase_confrontation | -1.72 | 3.92 | 76 | -0.44 | 0.662 |
| Audience_single | 2.78 | 2.81 | 76 | 0.99 | 0.325 |
| **Opposite-sex encounter** | | | | | |
| **Duration** | | | | | |
| Familiarity_unfamiliar | 14.01 | 42.61 | 14 | 0.33 | 0.747 |
| Sex_male | -35.87 | 30.02 | 14 | -1.19 | 0.252 |
| Door_open | 8.89 | 17.75 | 142 | 0.50 | 0.617 |
| **Pulse rate** | | | | | |
| Familiarity_unfamiliar | -0.07 | 0.17 | 14 | -0.45 | 0.659 |
| Sex_male | 0.09 | 0.10 | 14 | 0.91 | 0.377 |
| Door_open | -0.08 | 0.09 | 142 | -0.92 | 0.358 |
| **First to second pulse** | | | | | |
| Familiarity_unfamiliar | 0.01 | 5.73 | 14 | 0.00 | 0.998 |
| Sex_male | -6.16 | 4.05 | 14 | -1.52 | 0.151 |
| Door_open | 1.52 | 2.35 | 142 | 0.65 | 0.519 |
| **Same-sex encounters** | | | | | |
| **Duration** | | | | | |
| Familiarity_unfamiliar | 135.11 | 53.50 | 45 | 2.53 | **0.015** |
| Dyad_male-male | -93.40 | 38.61 | 2 | -2.42 | 0.137 |
| Door_open | 56.75 | 37.83 | 45 | 1.50 | 0.141 |
| Dominance index | -10.26 | 26.54 | 2 | -0.39 | 0.736 |
| **Pulse rate** | | | | | |
| Familiarity_unfamiliar | 0.03 | 0.24 | 45 | 0.14 | 0.890 |
| Dyad_male-male | 0.45 | 0.17 | 2 | 2.61 | 0.121 |
| Door_open | -0.23 | 0.17 | 45 | -1.38 | 0.175 |
| Dominance index | -0.19 | 0.12 | 2 | -1.61 | 0.248 |
| **First to second pulse** | | | | | |
| Familiarity_unfamiliar | -12.56 | 7.75 | 45 | -1.62 | 0.112 |
| Sex_male | -9.09 | 5.59 | 2 | -1.62 | 0.246 |
| Door_open | 2.76 | 5.48 | 45 | 0.50 | 0.617 |
| Dominance index | 6.11 | 3.85 | 2 | 1.59 | 0.253 |

**Table S3.**
Results of the bGLMM models testing the effects for drumming occurrence in predator, opposite-sex encounter and same-sex encounter experiments; SE: standard error; Bold: p < 0.05

| **No. of Drumming-call combinations** | | | | |
| --- | --- | --- | --- | --- |
| **Predator experiments (df = 190)** | | | | |
| Predictor | Estimate | SE | z-value | p-value |
| Condition_predator | 0.67 | 0.80 | 0.85 | 0.397 |
| Sex_female | 0.10 | 0.85 | 0.12 | 0.908 |
| Phase_confrontation | 1.51 | 0.79 | 1.90 | 0.058 |
| Audience_single | -0.09 | 0.64 | -0.14 | 0.890 |
| **Opposite-sex encounters (df = 124)** | | | | |
| Familiarity_unfamiliar | 2.15 | 0.96 | 2.23 | **0.026** |
| Sex_female | -1.44 | 0.83 | -1.73 | 0.084 |
| Door_closed | 0.27 | 0.49 | 0.54 | 0.586 |
| **Same-sex encounters (df = 106)** | | | | |
| Familiarity_unfamiliar | 2.43 | 1.54 | 1.58 | 0.115 |
| Dyad_female-female | -0.37 | 1.73 | -0.21 | 0.831 |
| Door_closed | -0.41 | 0.96 | -0.43 | 0.666 |
| Dominance index | 1.30 | 0.93 | 1.40 | 0.162 |

**Table S4.**Mean and standard deviation of the acoustic parameters for call types found mainly in our experiments; nomenclature based on Kobayasi et al. (2012) combined with Ter-Mikaelian et al. (2012)

| **Acoustic parameter** | **arched frequency modulated syllables** | **short bent upward frequency modulated syllables** | **upward sinusoidal frequency modulated syllables** |
| --- | --- | --- | --- |
|  | **alarm calls** | **contact calls** | **mating calls** |
| Time-related parameters | | | |
| C_Dur [ms] | 86.9 ± 55.4 | 30.8 ± 10.2 | 109.3 ± 50.5 |
| TimeminF0 [ms] | 63.7 ± 56.4 | 8.9 ± 5.6 | 19.0 ± 11.1 |
| TimemaxF0 [ms] | 40.6 ± 39.2 | 25.3 ± 11.3 | 92.9 ± 48.8 |
| Source-related parameters | | | |
| MinF0 [kHz] | 19.7 ± 3.4 | 26.2 ± 2.7 | 32.4 ± 4.9 |
| MaxF0 [kHz] | 25.0 ± 2.0 | 31.7 ± 2.8 | 48.3 ± 4.7 |
| BandF0 [kHz] | 5.4 ± 3.9 | 5.4 ± 2.4 | 15.9 ± 5.8 |
| MeanF0 [kHz] | 23.8 ± 1.3 | 28.6 ± 2.5 | 40.0 ± 4.0 |
| SDF0 [kHz] | 1.3 ± 1.2 | 1.8 ± 0.7 | 5.3 ± 2.1 |
| SlopeF0 [kHz/s] | 133.1 ± 105.1 | 399.4 ± 158.6 | 505.3 ± 297.8 |
| Filter-related parameters | | | |
| CoG [kHz] | 23.7 ± 3.1 | 39.9 ± 6.3 | 51.1 ± 3.4 |
| SD [kHz] | 4.0 ± 4.3 | 18.6 ± 4.0 | 18.6 ± 2.0 |
| Ske | 11.7 ± 9.8 | 1.3 ± 1.1 | 0.1 ± 0.3 |
| Kur | 400.2 ± 378.5 | 2.2 ± 5.5 | 0.4 ± 1.0 |
| Tonality-related parameters | | | |
| Voiced [%] | 96.5 ± 6.3 | 75.6 ± 16.5 | 50.1 ± 23.0 |
| Hnr [dB] | 24.0 ± 6.4 | 3.7 ± 2.8 | 2.1 ± 1.8 |
| Entropy [dB] | -4.2 ± 1.3 | -0.4 ± 0.5 | -0.2 ± 0.2 |
